# The effect of cranberry juice and a cranberry functional beverage on the growth and metabolic activity of selected oral bacteria

**DOI:** 10.1186/s12903-021-02025-w

**Published:** 2021-12-20

**Authors:** Paulina M. Nowaczyk, Joanna Bajerska, Małgorzata Lasik-Kurdyś, Elżbieta Radziejewska-Kubzdela, Artur Szwengiel, Małgorzata Woźniewicz

**Affiliations:** 1 Department of Sports Dietetics, Faculty of Health Sciences, Poznan University of Physical Education, Królowej Jadwigi 27/39, 61-871 Poznan, Poland; 2 Department of Human Nutrition and Dietetics, Faculty of Food Sciences and Nutrition, Poznań University of Life Sciences, Wojska Polskiego 31, 60-624 Poznań, Poland; 3 Department of Food Technology of Plant Origin, Faculty of Food Sciences and Nutrition, Poznań University of Life Sciences, Wojska Polskiego 31, 60-624 Poznań, Poland

**Keywords:** Cranberry, Functional food, Antimicrobial, Oral pathogens, Oral diseases

## Abstract

**Background:**

The oral microbiota is a significant risk indicator for oral diseases, such as dental caries and periodontal inflammation. Much attention is presently paid to the development of functional foods (e.g. beverages containing cranberry constituents, or foods containing probiotics) that may serve as adjuncts for oral disease treatments (e.g. periodontitis and caries). Cranberry fruit, due to its unique chemical composition and antimicrobial potential, is a possible ingredient of such foods. The study aimed to investigate the effects of cranberry juice (CJ) and a cranberry functional beverage (mixture of 80% v/v apple juice, 20% v/v cranberry juice, and 0.25 g/100 mL ground cinnamon; CFB) on the growth and metabolic activity of selected oral bacteria.

**Methods:**

Serial dilution pour plate method (SDPP) was used to examine the effect of CJ and CFB on the growth of *Actinomyces naeslundii*, *Streptococcus mutans,* and *Lactobacillus paracasei* subsp. *paracasei*. 48-h electrical impedance measurements (EIM) during the cultivation of *A. naeslundii* were applied to evaluate the utility of the method as a rapid alternative for the assessment of the antimicrobial potential of cranberry beverages.

**Results:**

The tested bacteria differed in their susceptibility to the antimicrobial action of CJ and CFB, with *L. paracasei* subsp. *paracasei* being least vulnerable to CFB (according to SDPP). Although CJ at a concentration of 0.5 mL/mL, showed a bactericidal effect on the growth of *S. mutans*, *A. naeslundii* was more sensitive to CJ (SDPP). Its inhibitory effect on *A. naeslundii* was seen even at concentrations as small as 0.03125–0.125 mL/mL (SDPP and EIM). On the other hand, *S. mutans* seemed to be more vulnerable to CFB than *A. naeslundii* (SDPP).

**Conclusions:**

CFB may be considered an adjunct in the treatment of oral diseases due to its action against selected oral pathogens, and not against the presumably beneficial *L. paracasei* subsp. *paracasei*. Bioelectrical impedance measurements appear to be a quick alternative to evaluating the antimicrobial activity of fruit beverages, but their utility should be confirmed with tests on other bacteria.

## Background

The pathogenic oral microbiota is a significant risk indicator for oral diseases, such as dental caries and periodontal inflammation [1]. Prevention and treatment of these diseases can include removing as many pathological microbiotas as possible using physical methods (tooth brushing, scaling, and root planning) or chemical methods (mouth rinses and antibiotic therapy). Some methods, such as antibiotic therapy, are questionable due to their possible side effects, such as the acquisition of resistance by oral pathogens or their negative effect on gastrointestinal tract microbiota. In recent years, the issue of mouthwash safety has also been raised. In particular, it has been suggested that regular long-term use of such items may lead to an overgrowth of pathogenic or resistant bacteria, eventually reducing the clinical efficacy of antibiotics [2–4]. Furthermore, the regular use of mouthwashes containing chlorhexidine or cetylpyridinium chloride is thought to contribute to an increased risk of hypertension (as a result of the destruction of oral microbes responsible for catalyzing the reduction of nitrate to nitrite) [2, 5–11] and to the development of prediabetes or diabetes [2]. Thus, much attention is presently being paid to the search for safe adjuncts for oral disease treatment. One solution may be found in so-called functional foods—items consumed as part of a regular diet and possessing health-related benefits that may contribute to diminishing the risk of specific chronic diseases [12]. In recent decades, cranberry fruit (*Vaccinium macrocarpon*) has received much attention regarding its possible use in the treatment of metabolic and oral diseases, including periodontal diseases [13–15]. The unique chemical composition of this fruit, including the presence of A-type procyanidins, makes it a potential ingredient of functional foods. It has previously been observed that cranberry compounds can inhibit the activity of *Streptococcus sorbinus* and *Streptococcus mutans* [15–20] and also possess antiadhesive potential against those bacteria, as well as against some periopathogens such as *Porphyromonas gingivalis* and *Fusobacterium nucleatum* [15, 21–25]; it also seems that they can restrain bacterial biofilm formation [14, 26–28]. It has been suggested that cranberry constituents can modulate the host's immune response in the course of periodontitis [27, 29–31]. Apart from oral health, cranberry beverages have also been studied concerning their possible benefits in modulating inflammation and oxidative status in overweight adults and patients with metabolic syndrome [32–34]. However, the use of cranberry in functional foods production has to date been limited due to its tart and astringent taste, which affect the taste and flavor of food items it enriches. Attempts have been made to overcome this disadvantage by adding artificial sweeteners, such as sucralose, aspartame, and acesulfame K. However, undesirable descriptors—such as sweet and bitter aftertaste, bitterness, and metallic flavor—are frequently reported for such beverages [35, 36]. In our previous study, we developed a functional beverage containing cranberry juice without any added sugars or artificial sweeteners, intended to possess a highly acceptable taste and other organoleptic features while being an effective and risk-free agent for supporting standard nonsurgical periodontal treatment. Apple juice—a highly acceptable, widely available, and relatively economical resource—was used as a base of the beverage; 100% cranberry juice was used as the main bioactive compound and the proportion used made up the highest sensory acceptable percentage of the cranberry juice in the beverage, based on our unpublished consumer sensory evaluation of four different variants of the beverage with cranberry juice; ground cinnamon was also used to enrich the taste. These considerations led to the following formula: 80% v/v apple juice (from the variety Antonówka Zwykła), 20% v/v of cranberry juice (*Vaccinium macrocarpon*), and 0.25 g/100 mL of ground cinnamon. This beverage, which we refer to as the cranberry functional beverage (CFB), was successfully introduced as an adjunct to standard periodontal therapy in patients with gingivitis. Full details can be found in our previous work [37].

For the present study, we intended to characterize the antimicrobial potential of CFB in vitro; we hypothesized that CFB possesses antimicrobial activity against selected oral pathogens (*Streptococcus mutans* and *Actinomyces naeslundii)* and does not affect the growth of the presumably beneficial *Lactobacillus paracasei* subsp. *paracasei*. We assessed the effects of CFB on the growth of these oral bacteria using the serial dilutions pour plate (SDPP) method, and we compared the activity of CFB with the activity of 100% cranberry juice (CJ). Furthermore, we attempted to verify the results obtained using SDPP via the quick alternative method of electrical impedance measurement (EIM) on one selected pathogen, *A. naeslundii*.

## Material and methods

### Antimicrobial agents

Cranberry juice (CJ) was obtained from *Vaccinium macrocarpon* fruits. The fresh fruits were washed, heated to 80 °C, and pressed in a Bucher press (Niederweningen, Switzerland). Immediately after cooling, the CJ was stored at − 20 °C until the microbiological tests were performed. The CFB was produced according to the above formula by the firm Polska Róża Ernest Michalski. The chemical composition and nutritional value of CJ and CFB are shown in Table 1.

**Table 1:** Chemical composition, acidity, and pH of cranberry juice (CJ) and cranberry functional beverage (CFB)

Characteristics	CJ	CFB
Glucose (g/L)	22.2 ± 0.4	26.5 ± 0.25
Fructose (g/L)	3.3 ± 0.1	73.6 ± 0.35
Sucrose (g/L)	≤ 2	6.2 ± 0.3
Vitamin C (mg/100 g)	2.4 ± 0.7	1.3 ± 0.35
Total polyphenols (g/L)	2.17 ± 0.06	3.22 ± 0.03
Anthocyanins (mg/100 mL)	5.4	0.7
Ash (g/L)	1.4	1.9
Total antioxidant activity (µM Trolox/mL)*	11.50 ± 0.43	6.84 ± 0.22
Antioxidant capacity (µM Trolox/mL)^#^	10.57 ± 0.24	2.64 ± 0.04
pH	2.5	3.5
Acidity (g/L)	17.68 ± 0.03	8.62 ± 0.04
Volatile acidity (g/L)	0.23 ± 0.02	0.10 ± 0.02

Results are presented as means ± SDs; *determined by the method utilizing 2,2’-azino-bis(3-ethylbenzothiazoline-6-sulphonic acid) free radical (ABTS), ^#^determined by the method utilizing 2,2-diphenyl-1-picrylhydrazyl free radical (DPPH)

### Phenolic compounds analysis

Reversed-phase (C18 column) ultra-high-performance liquid chromatography-electrospray ionization-mass spectrometry (RP-UHPLC-ESI–MS) analysis was performed using a Dionex UltiMate 3000 UHPLC (Thermo Fisher Scientific, Sunnyvale, CA, USA) coupled to a Bruker maXis impact ultrahigh resolution orthogonal quadrupole-time-of-flight accelerator (qTOF) equipped with an ESI source and operated in the positive- and negative-ion mode (Bruker Daltonik, Bremen, Germany). The RP chromatographic separation was achieved with a Kinetex™ 1.7 µm C18 100 Å, LC column 100 × 2.1 mm (Phenomenex, Torrance, CA, USA) according to Biesaga and Pyrzyńska [38]. The ESI–MS settings were previously described by Mildner-Szkudlarz et al. [39]. Molecular ions: [M + H]^+^ and [M-H]^−^ for phenolic compounds were extracted from full scan chromatograms (± 0.003 m/z) and peak areas were integrated with TASQ 2.1 (Bruker Daltonik, Bremen, Germany). The compounds present in each sample were identified based on the retention time of standard and/or molecular mass and structural information from the MS detector during MS/MS experiments. Additionally, the hydrolysis of samples was performed to confirm the presence of phenolic glycosides identified using MSMS spectra. The occurring of phenolic aglycones or increase in their concentration after hydrolysis proves the presence of glycosides. The samples were hydrolyzed with 1.2 M HCl for 2 h at 90 °C using the method described previously by Nuutilla et al. [40]. The tandem mass spectrometric data were used for searching molecular structure using CSI:FingerID (Friedrich Schiller University, Jena Germany), which combines fragmentation tree computation and machine learning [41, 42]. Limit of quantification (LOQ where S/N > 15) was determined for caffeic acid, chlorogenic acid, *p*-coumaric acid, sinapic acid, quercetin and it was not lower than 0.01 µg/mL.

The content of selected phenolic compounds of CJ and CFB is given in Table 2. The presented compounds were selected based on their antibacterial potential known from previous studies or their abundance in the tested beverages. In general, CJ was richer in phenolic compounds compared to CFB.

**Table 2:** Selected phenolic compounds of cranberry juice (CJ) and cranberry functional beverage (CFB)

Compound	Molecular Formula	MS precursor ion	MSMS ions	CJ (µg/100 mL)	CFB (µg/100 mL)
Quercitin pentoside^*^	C_20_H_18_O_11_	[M-H]^−^	151, 227, 243, 255, 271, 300	1962.9 ± 54.0	347.6 ± 10.7
Quercitin pentoside^*^	C_21_H_20_O_11_	[M-H]^−^	151, 179, 227, 255, 271, 300	542.6 ± 0.3	96.6 ± 0.9
Quercitin	C_15_H_10_O_7_	[M-H]^−^	107, 121, 151, 179, 227, 243	16.6 ± 0.1	< LOD
Procyanidin isomers^*^	C_30_H_26_O_12_	[M + H]^+^	123, 127, 139, 163, 287, 409	614.3 ± 11.0	628.1 ± 8.1
Epicatechin	C_15_H_14_O_6_	[M + H]^+^	123, 139, 207	945.0 ± 15.4	757.9 ± 12.9

Results are presented as means ± SDs; *calculated according to standard curves of appropriate aglycone; LOD level of detection

### Tested microorganisms

Strains of *Actinomyces naeslundii* (DSMZ 17,233), *Streptococcus mutans* (DSMZ 20,523), and *Lactobacillus paracasei* subsp. *paracasei* (DSMZ 4905) were used as test microorganisms. All strains were purchased from the Deutsche Sammlung von Mikroorganismen und Zellkulturen, Germany. These bacteria were cultured using the following media: Actinomyces Broth Vegitone for *A. naeslundii*; Casein-soy broth or agar with yeast extract for *S. mutans* (P-0236 or P-0237, BTL, Łódź, Poland), and MRS broth or agar for *L. paracasei* subsp. *paracasei* (CM0359 or CM0361, Oxoid, Hampshire, UK).

### Preparation of test cultures

For both experiments, liquid monocultures of the tested bacterial strains were prepared. The cultures for SDPP were prepared in standard glass tubes to a volume of 5 mL. The cultures for electrical impedance measurement were prepared in special 10-mL measuring tubes that were incubated in the automated microbiological growth analyzer (BacTrac 4100, Sy-Lab, Austria). A series of two-fold dilutions of CJ (0.50 mL/mL CJ1, 0.25 mL/mL CJ2, 0.125 mL/mL CJ3, 0.0625 mL/mL CJ4, 0.03125 mL/mL CJ5) or CFB (0.50 mL/mL CFB1, 0.25 mL/mL CFB2, 0.125 mL/mL CFB3) with sterilized liquid growth media were prepared and inoculated with the test microorganism. The applied inoculum constituted 5% for SDPP and 10% for impedance measurement of the volume of the entire culture. Aside from the test cultures enriched with CJ or CFB and inoculated with the test microorganisms, reference cultures (RC) inoculated with the test microorganisms, but not enriched with CJ or CFB, were also prepared.

### Serial dilutions pour plate (SDPP) method

To evaluate the inhibitory effects of CJ and CFB, the number of bacteria was determined at the 0 h time point and after 48 h of incubation at 37 °C. To that end, a volume of 0.1 mL of each liquid culture (after a series of decimal dilutions) was placed on sterile Petri dishes, covered with sterile agar medium; and incubated at 37 °C for 48 h. Colony forming units (cfu) were then counted, and dishes with cfu counts ranging from 30 to 300 were considered. The pour plate cultures were prepared in triplicate. The results of the experiment are presented as the mean number of cfu/mL of cultures after 48 h of incubation (Table 3). Further, the numbers of cfu/mL of each culture were log-transformed, and the differences (changes) between the log of the cfu/mL number at 48 h and 0 h in each culture were calculated.

**Table 3:** Number of cfu per 1 mL of reference and test cultures at 48 h

Antimicrobial agent	Concentration of CJ or CFB (mL/mL)	Culture symbol	*A. naeslundii*	*S. mutans*	*L. paracasei* subsp*. paracasei*
Reference culture	–	RC	6.57 × 10^7^	9.40 × 10^7^	2.80 × 10^8^
Cranberry juice	0.50000	CJ1	5.00 × 10^5***^	No growth	3.23 × 10^6***^
	0.25000	CJ2	7.43 × 10^7NS^	1.58 × 10^7***^	2.04 × 10^8**^
	0.12500	CJ3	1.93 × 10^7***^	1.45 × 10^8#^	4.46 × 10^8##^
	0.06250	CJ4	4.60 × 10^7**^	2.80 × 10^8###^	2.85 × 10^8NS^
	0.03125	CJ5	4.60 × 10^7***^	2.46 × 10^8###^	2.86 × 10^8NS^
Cranberry functional beverage	0.50000	CFB1	5.97 × 10^7NS^	6.40 × 10^7*^	1.45 × 10^8**^
	0.25000	CFB2	1.63 × 10^8##^	1.93 × 10^8##^	5.73 × 10^8##^
	0.12500	CFB3	5.07 × 10^8###^	2.23 × 10^8##^	2.34 × 10^8NS^

Values are means from three replicates. *, **, ***: Significantly lower than reference culture (*p < 0.05, **p < 0.01, *** *p* < 0.001). ^#, ##, ###^: Significantly higher than reference culture (^#^*p* < 0.05, ^##^*p* < 0.01, ^###^*p* < 0.001). CJ, cranberry juice; CFB, cranberry functional beverage; NS, not significantly different from reference culture

### Electrical impedance change measurements

The inhibitory effect of CJ and CFB against *A. naeslundii* was additionally verified by analyzing the electrical impedance changes in the growth medium during incubation. Such changes, caused by the metabolic activity of *A. naeslundii* during its growth in the medium containing CJ or CFB, were measured using an automated microbiological growth analyzer (BacTrac 4100, Sy-Lab, Austria). Special 10-mL measuring test tubes (Sy-Lab), equipped with four electrodes, were filled with 9 mL of growth medium containing CJ (0.50–0.03125 mL/mL) or CFB (0.50–0.125 mL/mL), and then inoculated with 1 mL inoculum of the tested bacteria. The measuring tubes were incubated at 37 °C in the analyzer’s thermostat. Changes in the electrical impedance were calculated using the formula:

$$y=\frac{\left({y}_{0}-{y}_{i}\right)}{{y}_{0}}\times 100\%$$

where *y*—is the change (expressed in %) in the electrical impedance of the growth medium, *y*_0_—is the value of electrical impedance at the beginning of culturing, and *y*_*i*_—is the value of electrical impedance at a common point of measurement (measured every 10 min).

The changes in electrical impedance caused by the bacterial metabolic processes can be presented as a curve that parallels the classic microbial growth curve with lag, logarithmic, and stationary phases [41–45]. To facilitate comparative analysis, we employed an impedance threshold of 2% of the changes and determined the parameter of impedance detection time (IDT).

### Statistical analysis

Data were analyzed using the *T*-test for independent variables, except for the comparison of IDT between CJ2 and RC, in which case the one-sample *T*-test was used. Statistical significance was set at *p* < 0.05. All analysis was performed using the StatSoft Statistica data analysis software system (version 13.1, 2016; www.statsoft.com).

## Results

### Actinomyces naeslundii

**Fig. 1:**
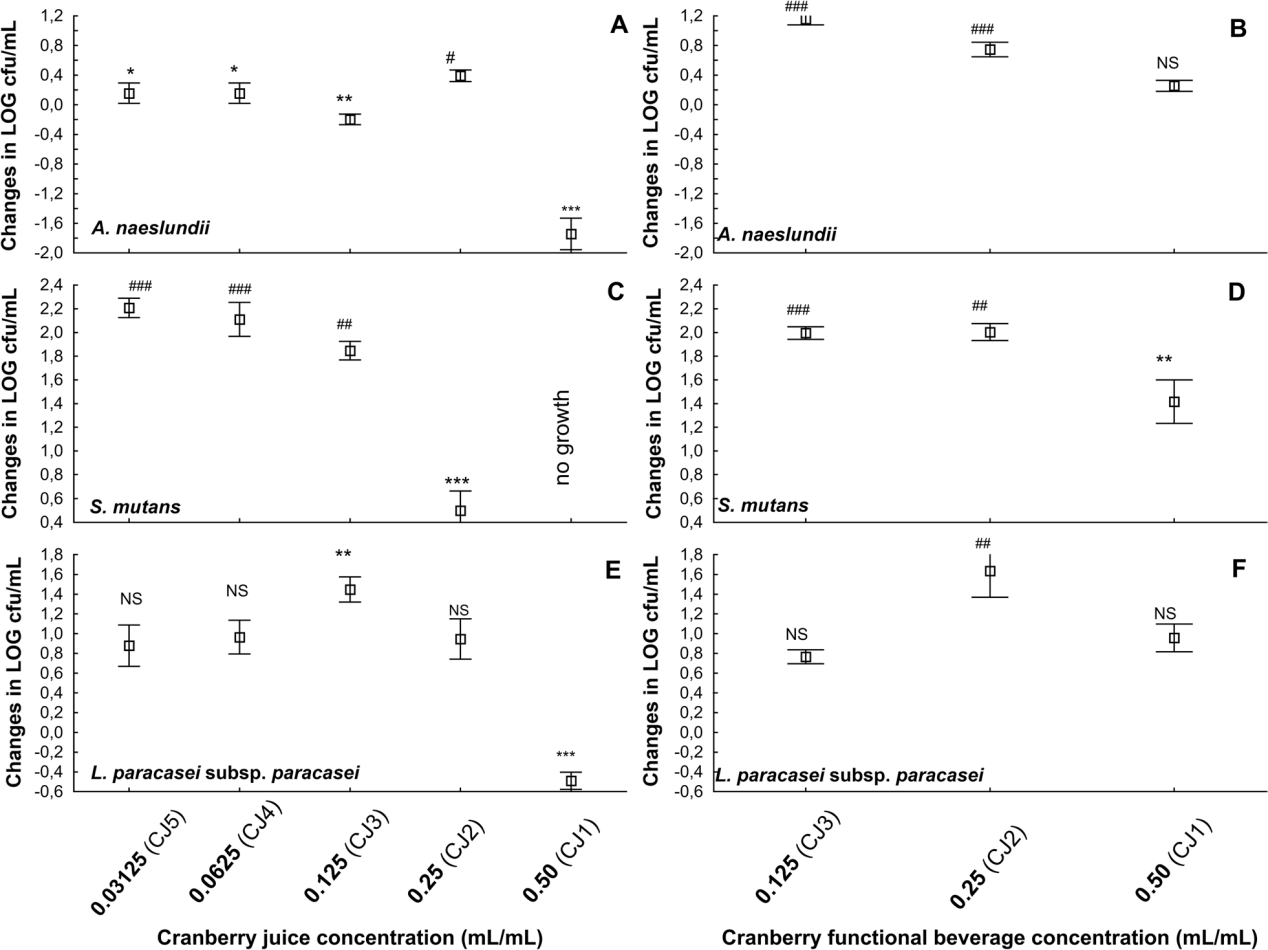
Changes in the number of cfu/mL between 0 and 48 h time points. **a**, **b** *A. naeslundii*, **c, d** *S. mutans*, **e**, **f** *L. paracasei* subsp. *paracasei*. Results are expressed as means ± SDs. *, **, ***: Significantly lower than reference culture (*p < 0.05, **p < 0.01, ***p < 0.001). ^#, ##, ###^: Significantly higher than reference culture (^#^*p* < 0.05, ^##^*p* < 0.01, ^###^*p* < 0.001). NS: not significantly different from reference culture. CJ, cranberry juice; CFB, cranberry functional beverage

The number of cfu/mL of *A. naeslundii* at 48 h was significantly lower in CJ1, CJ3, CJ4, and CJ5 than in the reference culture (RC; Table 3). No significant difference was found between CJ2 and RC. In the CFB2 and CFB3 cultures, the number of cfu/mL at 48 h was substantially higher than in RC. No difference was seen between CFB1 and RC. Compared to changes in the number of cfu/mL in RC—which illustrates the growth of the tested microorganism under optimal growing conditions—an inhibitory effect of CJ was observed at concentrations of 0.50 mL/mL (CJ1) and 0.125–0.03125 mL/mL (CJ 3–5); surprisingly, a slight stimulation of *A. naeslundii* growth was seen at 0.25 mL/mL (CJ2, Fig. 1a). At the concentration of 0.50 mL/mL CFB, the intensity of *A. naeslundii* growth was comparable to that in RC, while at 0.125–0.25 mL/mL CFB, an intensification was seen (Fig. 1b).

**Fig. 2:**
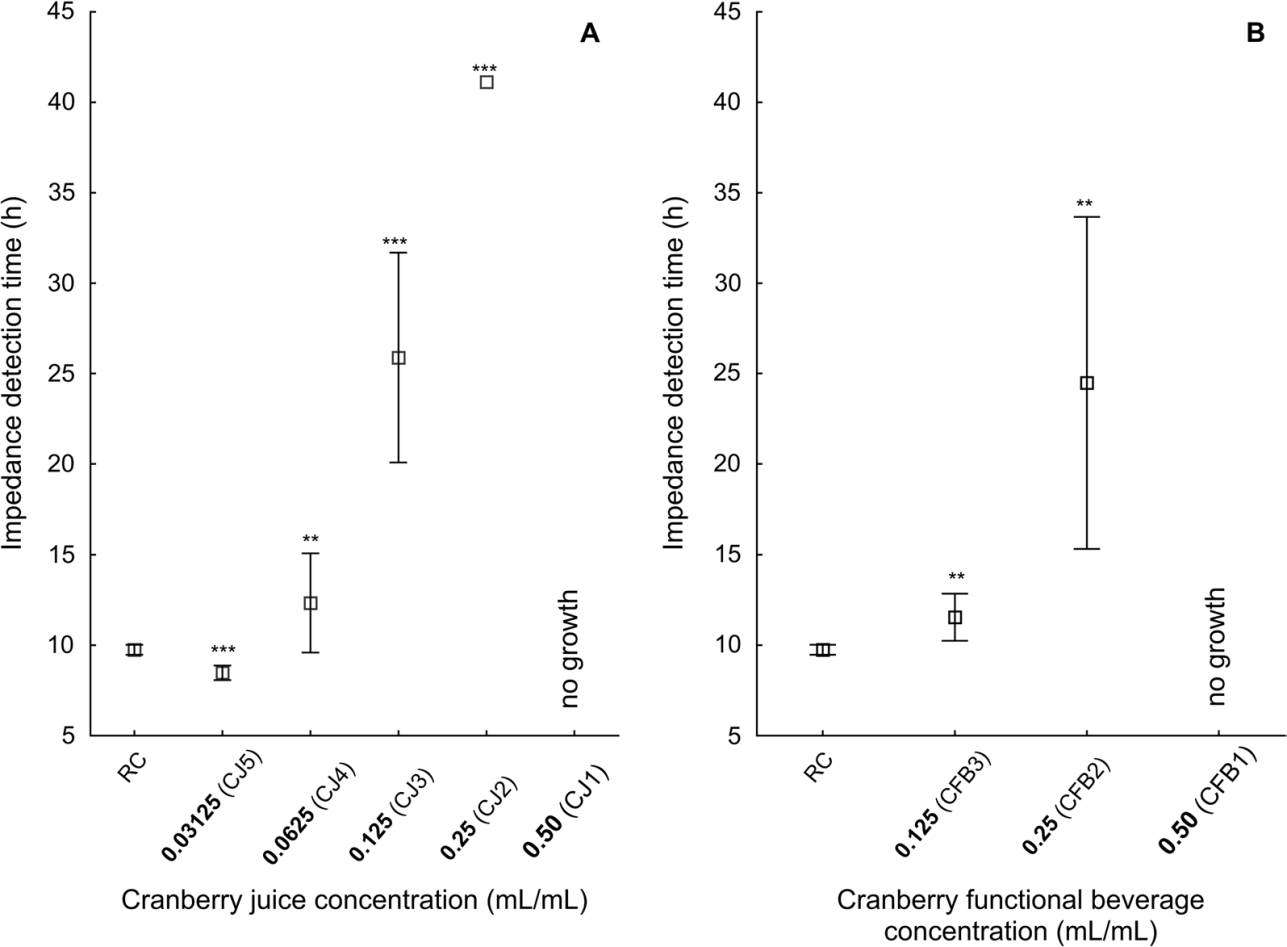
Impedance detection time of *A. naeslundii* in cultures with CJ (**a**) or CFB (**b**). Results are expressed as means ± SDs. *, **, ***: Significantly different from reference culture (*p < 0.05, **p < 0.01, ***p < 0.001). CJ: cranberry juice; CFB: cranberry functional beverage

Analyzing the changes in electrical impedance during incubation of the tested microorganisms an inhibiting effect in both cases (CJ as well as CFB) was observed. The significant differences in the value of impedance detection time (IDT) for all the tested juices concentrations were registered. In general, the higher was the concentration of CJ or CFB, the higher was the growth inhibition of the tested *A. naeslundii* (the longer was the IDT). In the highest concentration (0.50 mL/mL) a bactericidal effect of CJ was noted (Fig. 2a). The inhibition of *A. naeslundii* metabolic activity was noted in CJ2, CJ3, and CJ4 (as determined by the longer IDT in those cultures in comparison to RC). Similarly, at a concentration of 0.50 mL/mL of CFB, a bactericidal effect was seen (Fig. 2b). In CFB2 and CFB3, inhibition of *A. naeslundii* metabolic activity was also observed (IDT was significantly longer than in RC).

### Streptococcus mutans

No growth of *S. mutans* was observed at 48 h in CJ1 (0.50 mL/mL; bactericidal effect, Table 3). The number of cfu/mL was significantly lower in CJ2 than in RC. In subsequent cultures enriched with CJ, the number of cfu/mL was higher than in RC. Regarding the cultures enriched with CFB, lower numbers of cfu/mL than in RC were observed solely in CFB1, while these values for CFB2 and CFB3 were higher than in the RC. Compared to the changes in the number of cfu/mL in RC, an inhibitory effect of CJ was observed at a concentration of 0.5 and 0.25 mL/mL (CJ1 and CJ2), and of CFB at a concentration of 0.50 mL/mL (CFB1, Fig. 1c, d). In all succeeding cultures, an intensification of *S. mutans* growth was observed.

### *Lactobacillus paracasei* subsp. *paracasei*

At 48 h, the numbers of cfu/mL in CJ1 and CJ2 were significantly lower than in RC, and in CJ3 higher than in RC (Table 3). In the cultures enriched with CFB, significantly lower and significantly higher numbers of cfu/mL than in RC were observed in CFB1 and CFB2, respectively. Compared to the changes in the number of cfu/mL in RC, an inhibitory effect of CJ was observed only at a concentration of 0.50 mL/mL (CJ1), while an intensification in the growth of *L. paracasei* subsp. *paracasei* was observed at 0.125 mL/mL (CJ3; Fig. 1e). At no tested concentration did CFB inhibit the growth of *L. paracasei* subsp. *paracasei*, determined as changes in cfu/mL; at 0.25 mL/mL (CFB2), an intensification was observed in bacterial growth.

## Discussion

The results of our study support the hypothesis that cranberry functional beverage (CFB) possesses a certain antimicrobial activity against the oral pathogens *Actinomyces naeslundii* and *Streptococcus mutans*, but not against *Lactobacillus paracasei* subsp*. paracasei.* The bacteria *S. mutans* and *A. naeslundii* differ in their vulnerability to the inhibitory action of CJ and CFB. CJ possesses greater antimicrobial activity than CFB against the tested oral bacteria, including both oral pathogens and *L. paracasei* subsp. *paracasei*.

The evaluation of the antimicrobial effect of the tested beverages was performed using two methods: serial dilutions pour plate (SDPP) method and electrical impedance measurement. Similar studies on inhibitory and bactericidal activity evaluation [46–49] have indicated the usefulness of the impedimetric technique as a fast and precise test. Our results confirmed that bioelectrical impedance measurement can be an adequate and rapid method for determining the antimicrobial potential of fruit beverages against oral bacteria, at least in the case of *A. naeslundii*.

Each of the tested microorganisms has its own specific optimal growing conditions and specific effect on dental plaque biofilm development, as well as possessing characteristic adaptation mechanisms to changes in growth conditions, which under in vivo conditions concern the dental plaque microenvironment [50–52]. All these aspects affect the vulnerability of particular microorganisms to antimicrobial agents. Among the bacteria, we tested here, *Lactobacillus paracasei* subsp. *paracasei* proved to be relatively tolerant to the presence of CJ or CFB in the growth media. The role of the *Lactobacillus* genus in oral health is yet not clear. Studies of the involvement of *Lactobacillus* in the initiation and progression of dental caries have been carried out for decades, though without resolving the issue. The *Lactobacillus* genus contains about 80 species of bacteria. Although the total number of *Lactobacillus* spp. present grows with the progression of dental caries, it needs to be emphasized that some *Lactobacillus* species—such as *L. paracasei, L. plantarum*, *L. rhamnosus*, and *L. salivarius—*possess inhibitory potential against certain cariopathogens, like *S. mutans*. The inhibitory action seems to be greater in strains obtained from chronic periodontitis patients than in those from healthy volunteers [50, 53–55]. In view of this, the lack of strong antimicrobial potential of CJ or CFB against *L. paracasei* subsp. *paracasei* should not be perceived negatively. There is no data on the effect of cranberry components on oral lactobacilli species. The results of the studies on food microflora indicate nonetheless, that probiotic *Lactobacillus* species (*L. rhamnosus*), are less susceptible to the antimicrobial action of cranberry or blueberry phenolic compounds compared to foodborne pathogens (*E. coli*, *Listeria monocytogenes* or *Salmonella typhinurium*) [56, 57]. Phenolic compounds are generally known for their inhibitory action on bacteria growth. In recent years, however, it has been suggested that polyphenols can have even stimulatory effects on the growth of some bacteria, including species of lactic acid bacteria. The mechanism that gives *Lactobacillus* species greater resistance for phenolic compounds compared to the sensitivity of some oral pathogens (as shown in the current study) or food pathogens is unclear. Concerning lactic acid bacteria, they rely heavily on energy-transducing systems to survive in incessantly changing and often-hostile environments. Most of these metabolic energy-generating systems offer the prevention of a lethal decrease of the internal pH [58, 59]. The interaction between phenolic compounds and lactic acid bacteria is bidirectional, e.g. lactic acid bacteria can determine the bioavailability of polyphenols, and polyphenols can affect the growth of bacteria. The impact of phenolic compounds on lactic acid bacteria is determined by many factors, such as the structure of polyphenol, its concentration, bacterial species, and its growth phase, metabolic abilities, and adaptation response [60]. It is worth noting that apple juice, which constitutes 80 v/v% of CFB, has recently been proposed as a suitable medium for delivering certain strains of *Lactobacillus* (e.g. *L. paracasei*, *L. plantarum*) and producing potentially probiotic fruit juices [61]. It would be reasonable to consider enriching CFB with probiotic bacteria, to develop a product with even greater pro-health properties.

*Actinomyces naeslundii* is a gram-positive early colonizer of the dental plaque biofilm. It has two types of fimbriae: type 1 fimbriae facilitate its adhesion to acidic, proline-rich salivary proteins and statherin (which is present in the salivary pellicle). Type 2 fimbriae are associated with the attachment of *A. naeslundii* to the glycosidic receptors on epithelial cells, polymorphonuclear leukocytes, and oral streptococci. *A. naeslundii* associates and forms biofilms with *Streptococcus* and periodontal pathogens such as *Porphyromonas gingivalis* and *Fusobacterium nucleatum*, which have been known to induce alveolar bone resorption (in the course of periodontitis). Studies on animal models have reported alveolar bone destruction induced by *A. naeslundii* [62]. Some data are indicating the potential of a high molecular weight nondialysable material (NDM) from cranberry to inhibit the coaggregation of *A. naeslundii* with other periodontopathogenic microorganisms [21, 63, 64]. We have determined that *A. naeslundii* seems to be more sensitive than *S. mutans* to the presence of CJ in growth media, as the inhibitory effect was observed even at low concentrations of CJ. This was demonstrated by both the SDPP and electrical impedance measurement methods. As expected, CJ was a more potent inhibitor of *A. naeslundii* growth and metabolic activity than was CFB. The electric impedance measurements demonstrated a dose-dependent relation between the concentration of CJ or CFB in the growth medium and the degree of inhibition of the metabolic activity of *A. naeslundii*, as determined by IDT. Both tested agents exhibited a bactericidal effect at the highest concentrations, as measured by IDT; this is consistent with the results of the SDPP method, but only for the CJ-enriched culture.

*Streptococcus mutans* is a primary colonizer of dental plaque and is considered one of the primary causative agents of dental caries. This bacterium possesses a wide range of virulence factors, the most important of which include sucrose-independent and sucrose-dependent adhesion within the dental plaque (which plays a prominent role in initiating the changes in plaque ecology that lead to dental caries), acidogenicity, acid tolerance, and the production of a wide range of mutacins. All these features give *S. mutans* an ecological advantage over other microorganisms during the processes of dental biofilm formation and maturation [51, 65–71]. It has previously been observed in in vitro models that certain phenolic fractions of cranberry fruits (e.g. NDM and A-type cranberry proanthocyanidins) have the potential to inhibit the activity of glucosyltransferases and fructosyltransferases from *S. mutans*, and may interfere in sucrose-dependent and sucrose-independent adhesion of the bacteria. However, from the viewpoint of our experiment, the most likely mechanism of antimicrobial action of CJ and CFB against *S. mutans* would seem to be the interruption of adaptive processes that occurs at low pH; this is referred to as the acid tolerance response. This process involves the ability of *S. mutans* to maintain a transmembrane pH gradient, with the interior of the cell being more alkaline; this is achieved by upregulation of a proton-translocating F_1_F_0_-ATPase that extrudes H^+^ as the external environment becomes more acidic. Acid-tolerant growth is also associated with changes in metabolic pathways, such as the downregulation or upregulation of certain proteins [69]. Previous in vitro studies have indicated the potential of cranberry phenolics to affect the acid tolerance response of *S. mutans*, decreasing its acidogenic capabilities [17, 72]. The inhibitory effect of CJ on the growth of *S. mutans* was evident at high concentrations (0.25–0.50 mL/mL), whereas the effect was noted even at lower concentrations of CJ in the case of *A. naeslundii* (0.03125–0.1250 mL/mL). A concentration of 0.50 mL/mL of CFB was effective in inhibiting the growth of *S. mutans*, though not of *A. naeslundii*, as measured by the SDPP method alone.

The antimicrobial potential of cranberry and other fruit and vegetable juices against oral pathogens has been studied before. However, most of these studies focused on juice extracts or biochemical compounds derived from fruits and vegetables [73–75]. Studies of juices in the form they are habitually consumed are sparse [76]. In our study, we tested the beverages in a form that can be directly incorporated into the diet, and this should be seen as a strength of our approach. One limitation of our study is the use of planktonic monocultures of bacteria; the dental biofilm is generally less sensitive to antimicrobial agents than the planktonic form, and shows greater pathogenic synergism, and is harder to remove via physical or chemical agents [51, 77, 78]. For this reason, the results of this study cannot be taken to demonstrate the effects of CJ or CFB on dental plaque in vivo. In fact, the anticipation of the actual action of any antimicrobial agent in in vitro models is nearly impossible. Molecular techniques have identified over 700 bacterial species in the subgingival microbiota, but about 50–60% of these are uncultivable [79, 80]. Nonetheless, in our previous study, where gingivitis patients drank 750 mL of the tested CFB daily for eight weeks, we did note a reduction in the number of *S. mutans* in dental plaque, compared to patients drinking water. In that study, having in mind the relatively high content of sugars of CFB, and thus possible risk of dental caries development or progression, we evaluated the number of *S. mutans* as a CFB safety control. We demonstrated then, that the consumption of CFB improves gingival and plaque indices, without posing a risk of caries development [37]. This is in agreement with the results of the current study. It should be also mentioned that the low pH of CFB and high content of acids might pose a risk of dental erosion. This pathological process is defined as the partial demineralization of the tooth surface caused by repeated exposure to acid. The microorganisms are not involved [81, 82]. However, taking into account the high phenolic content of CFB and its antioxidant capacity, this beverage should be recommended as part of a varied and well-balanced diet in which the erosive potential of fruit and fruit drinks is balanced by the consumption of milk and yoghurt (or other dietary calcium sources) that can serve as a protective factor.

Another methodological issue is that the numbers of cfu in the cultures at 0 h were not standardized in the pour plate experiment. However, the calculations allowed us to compare the changes in the number of cfu between the cultures enriched with CJ or CFB and the reference cultures.

## Conclusions

In conclusion, the tested bacteria differed in their susceptibility to the antimicrobial action of CJ and CFB, with *L. paracasei* subsp. *paracasei* being, as expected, the least vulnerable to CFB. Although CJ at a concentration of 0.5 mL/mL showed a bactericidal effect on the growth of *S. mutans*, *A. naeslundii* proved more sensitive to the presence of CJ in the growth medium, as demonstrated by the inhibitory effect of CJ on *A. naeslundii*, which was seen even at concentrations as low as 0.03125–0.125 mL/mL. *S. mutans* seemed to be more vulnerable to CFB than *A. naeslundii.* The results allow us to state that CFB may be further investigated as a possible safe adjunct in oral disease treatment on account of its action against selected oral pathogens, and not against the presumably beneficial *Lactobacillus paracasei* subsp. *paracasei.* In addition, bioelectrical impedance measurement appears to be a rapid alternative method for evaluating the antimicrobial activity of fruit beverages, but its utility should be confirmed with tests on other bacteria.

## Data Availability

The datasets generated and analysed during the current study are available from the corresponding author on reasonable request.

## Abbreviations

CFB: Cranberry functional beverage
SDPP: Serial dilutions pour plate
EIM: Electrical impedance measurements
CJ: Cranberry juice
LOD: Level of detection
RC: Reference culture
cfu: Colony forming units
IDT: Impedance detection time
